# Vitamin D Deficiency in Medical Patients at a Central Hospital in Malawi: A Comparison with TB Patients from a Previous Study

**DOI:** 10.1371/journal.pone.0059017

**Published:** 2013-03-28

**Authors:** Yamikani Mastala, Phempo Nyangulu, Rodrick V. Banda, Bongani Mhemedi, Sarah A. White, Theresa J. Allain

**Affiliations:** 1 Department of Medicine, College of Medicine, University of Malawi, Blantyre, Malawi; 2 Department of Community Health, College of Medicine, University of Malawi, Blantyre, Malawi; Institut de Pharmacologie et de Biologie Structurale, France

## Abstract

**Objectives:**

To determine the prevalence of vitamin D deficiency (VDD) in adult medical, non-tuberculous (non-TB) patients. To investigate associations with VDD. To compare the results with a similar study in TB patients at the same hospital.

**Design:**

Cross-sectional sample.

**Setting:**

Central hospital in Malawi.

**Participants:**

Adult non-TB patients (n = 157), inpatients and outpatients.

**Outcome Measures:**

The primary outcome was the prevalence of VDD. Potentially causal associations sought included nutritional status, in/outpatient status, HIV status, anti-retroviral therapy (ART) and, by comparison with a previous study, a diagnosis of tuberculosis (TB).

**Results:**

Hypovitaminosis D (≤75 nmol/L) occurred in 47.8% (75/157) of patients, 16.6% (26/157) of whom had VDD (≤50 nmol/L). None had severe VDD (≤25 nmol/L). VDD was found in 22.8% (23/101) of in-patients and 5.4% (3/56) of out-patients. In univariable analysis in-patient status, ART use and low dietary vitamin D were significant predictors of VDD. VDD was less prevalent than in previously studied TB patients in the same hospital (68/161 = 42%). In multivariate analysis of the combined data set from both studies, having TB (OR 3.61, 95%CI 2.02–6.43) and being an in-patient (OR 2.70, 95%CI 1.46–5.01) were significant independent predictors of VDD.

**Conclusions:**

About half of adult medical patients without TB have suboptimal vitamin D status, which is more common in in-patients. VDD is much more common in TB patients than non-TB patients, even when other variables are controlled for, suggesting that vitamin D deficiency is associated with TB.

## Introduction

Vitamin D is known to have widespread actions throughout the body, including the immune system [1]. Much attention has focussed on vitamin D deficiency and susceptibilty to mycobacterial infection. A high prevalence of vitamin D deficiency has been found in cross sectional studies among TB patients in different parts of the world [2]–[6]. The mechanisms underlying this association are partially understood [7]–[11], but despite known effects of vitamin D on immunity to TB, studies of vitamin D as adjunctive therapy for TB treatment have had mixed results [12]–[16].

Risk factors for vitamin D deficiency vary depending on the setting and the population studied. Few studies have examined risk factors for vitamin D deficiency in African populations living in Africa. Potential risk factors include poor dietary intake of vitamin D, medications such as anticonvulsants and TB drugs, lack of sunlight exposure, dark skin pigmentation, extremes of age and obesity [17]. Vitamin D deficiency is also common in patients with HIV infection and may be worsened by antiretroviral treatment (ART) [18], [19]. Vitamin D deficiency has been linked to a wide range of health outcomes including cardiovascular disease, diabetes, cancer, autoimmune diseases and both cardiovascular and all-cause mortality [1]. Thus patients with low levels of 25(OH)D might be over-represented on medical wards and clinics.

We have previously demonstrated one of the highest reported prevalences of hypovitaminosis D (serum 25-hydroxyvitamin D [25OHD] level ≤75 ng/mL) and vitamin D deficiency (VDD, 25OHD level ≤50 ng/mL) in 161 adult TB patients in Malawi; hypovitaminosis D was found in 74.5%, VDD in 42.2%, and severe vitamin D deficiency (sVDD, 25OHD level, ≤25 ng/mL) in 13.0% [3]. This group of patients had multiple risk factors for vitamin D deficiency, including poor nutrition, HIV infection and pigmented skin. However, that study had no comparator group of non-TB patients, so it is not known whether a high prevalence of vitamin D deficiency applies in general to sick Malawian adults, who share common risk factors, or whether patients with TB differ from patients with non-TB diagnoses with respect to vitamin D levels. In the current study, we sought to determine the prevalence of vitamin D deficiency in adult non-TB patients seen at the same tertiary hospital. The secondary objective was to compare the results to the 2008 study in TB patients, at the same hospital. We also sought to identify which of the following are predictors of vitamin D deficiency and 25OHD level: HIV status, nutrition, antiretroviral drug use, gender, age and in/out patient status.

## Methods

This was a cross sectional descriptive study which took place at Queen Elizabeth Central Hospital (QECH) in Blantyre, Malawi in June and July 2010. QECH provides secondary care for a population of approximately 1 million and is the main referral hospital in the southern region of Malawi. Patients, aged over 18 years old, admitted in the medical wards for conditions other than TB or treated as outpatients for conditions other than TB, were eligible to be recruited. Exclusion criteria were a current diagnosis of TB, suspected TB (chronic cough of more than 3 weeks duration and/or constitutional symptoms of unexplained fevers, night sweats or weight loss and failure to respond to appropriate anti-bacterial antibiotics), and patients undergoing investigations for extrapulmonary TB. Cases were selected so the sample had similar characteristics in terms of age (within 5 years), gender and inpatient/outpatient status to the TB patients studied 2 years previously. Samples were collected during a 1 month period at the same time of year.

Using a standardised questionnaire (Appendix S1) information on age, gender, life style (time spent outdoors), religion, socioeconomic status, race/ethnicity, education level, marital status, diet, daily sun exposure, medical diagnosis, length of hospital stay, HIV status and medication, including ARV drugs was collected from the patient, their guardian and their medical record. Weight and height were measured. The medical diagnosis was the working diagnosis of the clinician in charge of the patient at the time of data collection. Diet questions covered consumption of milk products, meat, fish, eggs and margarine.

Blood samples were collected for 25(OH)D levels, centrifuged soon after collection and serum stored at −20°C. In both studies a 25-Hydroxy Vitamin D_3_ enzyme immune assay (EIA) (Immunodiagnostic Systems Ltd, Boldon, UK) was used to quantify 25(OH)D in serum samples. For each cohort the assays were carried out under the same conditions, in the same laboratory, with the same supervisors. Calibrations and controls provided by the manufacturer were used. Technical details of the assay are available at http://www.idsplc.com/en-gb/products/25-hydroxy-vitamin-d-eia-ac-57f1.

### Statistical Analysis

To compare the prevalence of vitamin D deficiency between in-patients and out-patients, and to identify predictors of vitamin D deficiency, Fisher's exact test, Pearson's Chi squared test, unpaired Student's t-test, and simple linear regression were used. Multiple logistic regression was used to compare TB and non-TB patients in terms of hypovitaminosis, VDD and sVDD with HIV status, in-patient status and BMI as potential risk factors. A sample size of 160 was aimed for, to match the sample of TB cases studied in 2008. A 5% significance level was used to declare significance. Analyses were performed using Stata version 12.

### Ethics Statement

The project proposal was reviewed and approved by the College of Medicine Research and Ethics Committee (COMREC), protocol number P03.10.906. Patients or guardians provided written informed consent at study enrolment.

## Results

One hundred and fifty seven patients were recruited, of whom 101 were in-patients. All subjects were black Malawians. The diagnoses in these patients were broadly similar to the case mix on the medical wards and clinics at QECH (excluding TB). The commonest diagnoses were bacterial pneumonia, heart failure and stroke in in-patients and upper respiratory tract infection and musculoskeletal problems in out-patients.

The mean 25(OH)D level in the study sample was 84.2 nmol/L (SD 40, range 77.9–90.6). Hypovitaminosis (≤75 nmol/L) was noted in 75 (47.8%) and vitamin D deficiency (≤50 nmolL) in 26 (16.6%) of patients. There was no case of severe vitamin D deficiency (≤25 nmol/L). Table 1 summarises 25(OH)D levels and vitamin D status according to social, demographic and clinical characteristics.

**Table 1:** Vitamin D levels and vitamin D status by social, demographic and clinical characteristics of patients.

VARIABLE	CATEGORY	FREQUENCY n (%)	Mean (95% CI) vitamin D level (nmol/L)	p for t test/ANOVA	Normal vit. D >75 nmol/L, n %	Hypovit. D ≤75nmol/L, n %	VDD ≤50nmol/L n %	p for VDD X^2^/Fishers exact test
GENDER	Male Female	83 (53) 74 (47)	82 (74–86) 87 (77–97)	0.39	40 (48.2) 42 (56.8)	43 (51.8) 32 (43.2)	14 (16.9) 13 (17.6)	0.55
AGE, years	18–30 31–50>50	58 (37) 65 (41) 34 (22)	90 (79–102) 81 (71–90) 82 (69–94)	0.38	30 (51.7) 32 (49.2) 20 (58.8)	28 (48.3) 33 (50.8) 14 (41.2)	8 (13.8) 9 (13.8) 10 (29.4)	0.15
BMI, Kg/m2	<20 20–25 >25	62 (39.5) 75 (47.8) 20 (12.7)	80 (69–91) 82 (75–90) 106 (84–126)	0.034	27 (43.5) 38 (50.7) 17 (85.0)	35 (56.5) 37 (49.3) 3 (15.0)	14 (22.6) 10 (13.3) 2 (10)	0.008
HIV status	Reactive Non Reactive Unknown	51 (32.5) 36 (22.9) 69 (43.9)	84 (70–97) 82 (74–90) 86 (76–95)	0.91	21 (41.2) 22 (61.1) 38 (55.1)	30 (58.8) 14 (38.9) 31 (44.9)	7 (13.7) 4 (11.1) 16 (23.2)	0.74
PATIENT status	In-patients Out- patients	101 (64.3) 56 (35.7)	80 (71 –88) 93 (85–101)	0.0005	42 (41.6) 40 (71.4)	59 (58.4) 16 (28.6)	23 (22.8) 3 (5.4)	0.005
ART use (of HIV R, n = 51)	On ART Not on ART	37 (72.5) 14 (27.5)	78 (62–94) 104 (77–131)	0.09	11 (29.7) 11 (78.6)	26 (70.3) 3 (21.4)	7 (18.9) 0	0.048
Vitamin D food source frequency	weekly <weekly	59 (37.6) 98 (62.4)	91.6 (82–101) 79.9 (72–88)	0.07	37 (62.7) 45 (45.9)	22 (37.3) 53 (54.1)	5 (8.5) 21 (21.4)	0.03

25(OH)D level, or the presence of vitamin D deficiency, were associated with low BMI, in-patient status, taking a dietary source of vitamin D less than once per week and ART use (Table 1). There was a weak positive correlation between BMI and 25(OH)D level, r^2^ = 0.028, p = 0.035. VDD was significantly more prevalent in those with lower BMI (Table 1). Mean 25(OH)D D level was significantly lower in inpatients than in outpatients (79.6 (SD 44.0, range 27–303) nmol/L and 92.6 (SD 29.7, range 34–200) nmol/L respectively) and VDD was more common in in-patients (22.8% vs 5.4%; X^2^ = 7.9, p = 0.005, df = 1). This was despite in-patients having a significantly higher BMI (mean BMI in-patients 22.3, 95% CI 21.2–23.4 vs out-patients 20.2, 95% CI 19.4 vs 21.0, p = 0.0035). Ninety eight patients stated that they ate any source of vitamin D less than weekly. Such patients had lower mean 25(OH)D levels (79.9 vs 91.6 nmol/L, t = 1.8, p = 0.07) and were significantly more likely to have VDD (21.4% vs 8.5%; p = 0.03). The association between dietary intake of vitamin D source food and VDD was independent of BMI. Alcohol consumption, marital status and smoking were not predictors of vitamin D status.

Of 51 HIV positive patients 38 were on ART. All of those were on first line regimen of a non nucleoside reverse transcriptase inhibitor (NNRTI, usually nevirapine) and 2 nucleoside reverse transcriptase inhibitors (NRTIs), in accordance with local guidelines. Duration of ART use was not collected. The mean serum 25(OH)D level was lower in those taking ART than in those not on ART (77.9 (SD 47.6, range 33–303) and 104.4 (SD44.8, range 56–205) nmol/L, respectively), although this was not statistically significant (p = 0.09). Vitamin D deficiency was more common in those taking ART (18.4% vs 0%). The difference in vitamin D status between ART users and non-users was not accounted for by differences in BMI or in-patient status.

Serum vitamin D levels and the prevalence of VDD did not differ with any of the other variables investigated.

Comparison of these patients with TB patients at the same hospital in 2008(3) is summarised in Table 2. In both studies, samples were collected over 4 weeks in the Winter months of June and July. By design age, gender and in-patient status were similar in the 2 cohorts, but TB patients were, on average 4 years younger, which was statistically significant (t = 3, p = 0.013). TB patients had lower mean 25(OH)D levels, (59.7 vs. 84.2 nmol/L, p<0.0001) and a higher prevalence of hypovitaminosis D (74.5 vs 47.8%, p<0.0001), VDD (42.2 vs 16.6%, p<0.0001) and severe VDD (11.2% vs 0, p<0.0001). TB patients also had lower BMI and were more likely to know their HIV status (Table 2). If the HIV status was known TB patients were more likely than non-TB patients to be HIV reactive. Information on ART use is not available for the TB patients, although at the time of that survey very few newly presenting, HIV positive TB patients were on ART.

**Table 2:** Comparisons of TB patients and non-TB patients.

Parameter	TB patients	Non TB patients	p-value
n	161	157	
Age in years mean (range)	35.1 (18–73)	38.9 (18–80)	t = 3, df = 316 p = 0.01
Gender n (% male)	82 (51.0)	83 (52.9)	Fisher's exact test p = 0.73
BMI Kg/m2 mean (95% CI)	19.5 (19–20)	20.9 (20.2–21.6)	t = 3, df = 316 p = 0.001
HIV status n (%) Reactive Non-reactive Unknown	106 (65.8) 23 (14.3) 32 (19.9)	51 (32.5) 36 (22.9) 69 (43.9)	Fisher's exact test p<0.0001
HIV- known status Reactive Non-reactive	106 (82.2) 23 (17.8)	51 (59.1) 36 (40.9)	Fisher's exact test p = 0.003
**Vitamin D level**			
nmol/L mean (95% CI)	59.7 (55.3–64.1)	84.2 (77.9–90.6)	t = 6.328 df = 316 p<0.0001
**Vitamin D status**			
hypovitaminosis D ≤75nmol/L n(%)	120 (74.5)	75 (47.8)	Fisher's exact test p<0.0001
VDD* ≤50nmol/L n(%)	68 (42.2)	26 (16.6)	Fisher's exact test p<0.0001
sVDD* ≤25nmol/L n (%)	18 (11.2)	0	p<0.0001

* VDD =  vitamin D deficiency, sVDD = severe vitamin D deficiency.

In multivariable analysis of the combined data set having TB (OR 2.59, 95%CI 1.51–4.42, p<0.0001) and being an in-patient (OR 3.02, 95%CI 1.72–5.30, p<0.0001) were independent predictors of hypovitaminosis D. The same variables were also significant independent predictors of VDD (TB; OR 3.61, 95%CI 2.02–6.43, p<0.0001 and in-patient status; OR 2.70, 95%CI 1.46–5.01, p<0.0001). There were no patients with sVDD in the non-TB cohort so the analysis could not be carried out.

## Discussion

We have demonstrated that inadequate serum 25(OH)D levels are common among adult medical in-patients; 58% have hypovitaminosis D (≤75 nmol/L) and 23% have vitamin D deficiency (≤50 nmol/L). The commonest diagnoses among these patients were cardiac disease, particularly heart failure, cerebrovascular disease and non-tuberculous infections including pneumonia. Low levels of vitamin D have been associated with heart failure and with worse outcomes in heart failure patients, including mortality [20]. The mechanisms underlying this association are considered to be multifactorial and reflect the diverse actions of vitamin D. It is suggested that vitamin D deficiency may be more common in heart failure patients due to increased age, renal impairment, poor sun exposure or obesity. Vitamin D deficiency is also associated with risk factors for heart disease such as diabetes, hypertension, vascular and myocyte dysfunction [21]. However, a recent meta-anlysis showed that vitamin D supplements did not improve cardiovascular outcomes [22]. Similarly, stroke risk is associated with vitamin D deficiency [23] and stroke severity may be influenced by vitamin D status [24]. Hence, high levels of vitamin D deficiency would be expected among a group of patients with cardiac and cerebrovascular disease. Most of our patients had a hospital stay period of less than one week which is not enough time for vitamin D stores to get depleted. Furthermore, there was no association between hospital stay period and VDD, so it is unlikely that the low vitamin D levels are a consequence of the admission to hospital. There are no published studies of the association of vitamin D and heart failure or stroke in Africa.

There are no available data on serum 25(OH)D levels in healthy adult Malawians, so we do not know whether the observed prevalence of vitamin D deficiency (23% in in-patients and 5% in out-patients) or hypovitaminosis D (58% in in-patients and 29% in out-patients) is higher than in the general population. There are few published studies of vitamin D levels in community samples from other African countries. A study from Guinea-Bissau, in West Africa, found a prevalence of hypovitaminosis D of 39% and VDD of 13% in a randomly selected sample of 494 adults [6]. A study of 50 healthy, HIV negative Ugandan adults found VDD in 20% [2]. These study populations are culturally and nutritionally diverse and of limited relevance to the Malawian population. Out-patients are more likely than in-patients to resemble the general public, and our results showing lower mean vitamin D levels in in-patients than out-patients suggest that our in-patients have lower levels than the general Malawian population.

The risk factors for VDD were lower BMI, a diet lacking vitamin D food sources, being an in patient and ART use. The relationship with BMI is interesting. Most studies from developed countries have found that high BMI is a risk factor for low serum vitamin D, presumably because vitamin D is a fat soluble vitamin and a high volume of adipose tissue acts as a sump for vitamin D. In this study, the studies from Tanzania [5], Uganda [2] and our previous study of TB patients [3] *low* BMI was a risk factor for VDD. In our study this was independent of dietary intake of vitamin D food sources. The relationsip with BMI in our patients may be explained by the very low BMIs in our patients. Patients with very little adipose tissue are unable to store vitamin D, so they have no reserves when external sources are lacking. The relationship with dietary intake, particularly fish, is expected and has been described before in Tanzanian patients [5]. There was no association with HIV status, an observation that was also made in Uganda [2] and Tanzania [5], however, among those who were HIV reactive, ART use was associated with lower 25(OH)D levels. Although ART drugs may alter vitamin D levels, eg through altered metabolism of vitamin D, there are a number of potential causative mechanisms. Among HIV positive patients VDD is associated with lower CD4 cell counts. We do not know the CD4 counts of our patients, neither do we know the duration of their ART use. Similarly, there are other potential risk factors for VDD, including renal function, that were not measured in our patients.

Another important finding of this study is the difference in vitamin D status in general medical patients compared with TB patients. The principal aim of this study was to provide a comparator group for the previous study of TB patients. We hypothesised that rates of vitamin D deficiency would be higher in TB patients than in non-TB patients and this was confirmed. In multivariate analysis having TB was independently associated with hypovitaminosis D, VDD and severe VDD. This is consistent with vitamin D deficiency having a specific role in increasing risk of TB in Malawian patients, which agrees with current thinking on TB risk and vitamin D deficiency [12].

There are some weaknesses of our study. We do not have a comparison group of healthy Malawian subjects so we do not know how much our sick adults differ from healthy adults. HIV status was unknown in 44% and some of the sub-group analyses eg among ART users lacked power to detect significant differences so important associations may have been missed. The subjects in the current study were, on average 4 years older than the TB patients. Although statistically significant this age difference is unlikely to be clinically important in terms of age related variation in vitamin D levels. The two studies were carried out 2 years apart and in our rapidly changing society unmeasured health and economic factors may have differed between the groups. We were unable to measure other biochemical variables such as calcium and renal function, which are related to vitamin D metabolism and would have been helpful in aligning the vitamin D levels measured to physiological and pathological variation in our subjects. Pneumonia was diagnosed in 11/157 (7%) of the non-TB patients. Any patient that was a potential TB suspect according to national criteria (cough of >3 weeks duration, constitutional symptoms of weight loss or night sweats and failure to respond to anti-bacterial antibiotics) was excluded, but it is still possible that some patients within the non-TB group were misclassified.

## Conclusion

In summary, these data add to accumulating evidence that vitamin D deficiency, a preventable health problem, is common among medical patients. This is the first report of 25(OH)D levels in medical patients in Africa. Furthermore, the even higher rates of vitamin D deficiency observed in Malawian adults with TB point to a specific association between TB and vitamin D deficiency, and that inadequate circulating 25(OH)D levels are not just a general phenomenon associated with chronic ill health or acute illness. These findings raise the challenging question about what should be done about this problem? Further studies are needed to determine the prevalence of and risk factors for vitamin D deficiency in the general population and specific disease states. It is important to examine whether vitamin D deficiency is a cause of ill health, or a consequence of it. Even though studies of vitamin D treatment in TB have had variable outcomes, vitamin D supplementation may have other benefits related to its physiological role, such as preventing the development of bone disease, infections, vascular diseases, autoimmune diseases and cancer.

## Supporting Information

Appendix S1 (DOCX)
