# Supplementary material for: Vitamin D Deficiency in Medical Patients at a Central Hospital in Malawi: A Comparison with TB Patients from a Previous Study
Source: PLoS One. 2013 Mar 28;8(3):e59017. doi: 10.1371/journal.pone.0059017 (PMC3610862; doi:10.1371/journal.pone.0059017)
Supplement: Appendix S1 — (DOCX) [file pone.0059017.s001.docx]

**APPENDIX**

**English questionnaire**

DEMOGRAPHIC DATA

Date of Interview

|  |  |  |
| --- | --- | --- |

Signature of interviewer: _________________

1. ID number……………
2. Age______________
3. Sex:
4. Male
5. female
6. Location
7. District
8. Tribe:
9. Lomwe
10. Chewa
11. Tumbuka
12. Ngoni
13. Yao
14. Sena
15. Others (specify)________________________

7. Patient skin color description

1. Dark
2. light

8. Education:

1. None
2. std 1-5
3. std 6-8
4. Secondary school

5. Tertially education

9. Occupation:

1. Employed
2. Unemployed
3. Self-employment
4. housewife

Others (specify) _____________________________________________________________________________________________________________

HEALTH INFORMATION

10) What sort of disease do you have?

11) Date of Diagnosis_________________________________________________________________

Question 14: in patient only

12) Date of admission________________

13) How many times have you been admitted because of the disease?

1. Once
2. Twice
3. Others(specify)___________________

14) What is your HIV sero-status?

1. Unknown
2. Negative
3. Positive

15) If HIV positive, are you on ARVs?

1. Yes

2. No

16) If yes, what sort of ARVs and for how long have you been on ARVs?__________________________________________________________________

17) What other medication/drugs are you on?

______________________________________________________________________________________________________________

Question 17: In-patients only

18) During the day how much time do you spend in the sun?

1. None
2. < 30 minutes
3. >30-60 minutes>
4. >1hr
5. others (specify)__
6. Question 18 -20 for out patients only

19. What activities do you do while you are exposed to the sun?

________________________________________________________________________

_________________________________________________________________________

_________________________________________________________________________

20. How much time do you spend on the sun while doing each of the mentioned activity per day?

_________________________________________________________________________

_________________________________________________________________________

21. How much time do you spend on the activity / week?

_________________________________________________________________________

___________________________________

____________________________________

22) Patient weight/kg__________________

23) Height__________

24) Measured Vitamin D levels

_______________

25) FOOD FREQUENCY QUESTIONNAIRE

| Type of food | How often do you eat … per week |
| --- | --- |
| Egg |  |
| Liver |  |
| Beef |  |
| Margarine fortified |  |
| Cat fish- Mlamba |  |
| Cat fish-kampango |  |
| Pork fat(lard) |  |
| Butter |  |
| Cheese |  |
| Milk |  |

26) Marital status

27) Type of fish often eaten

28) Alcohol intake

29) Smoking

30) Religion
